# Supplementary figures and images for: Ccq1–Raf2 interaction mediates CLRC recruitment to establish heterochromatin at telomeres
Source: Life Sci Alliance. 2021 Sep 7;4(11):e202101106. doi: 10.26508/lsa.202101106 (PMC8424379; doi:10.26508/lsa.202101106)

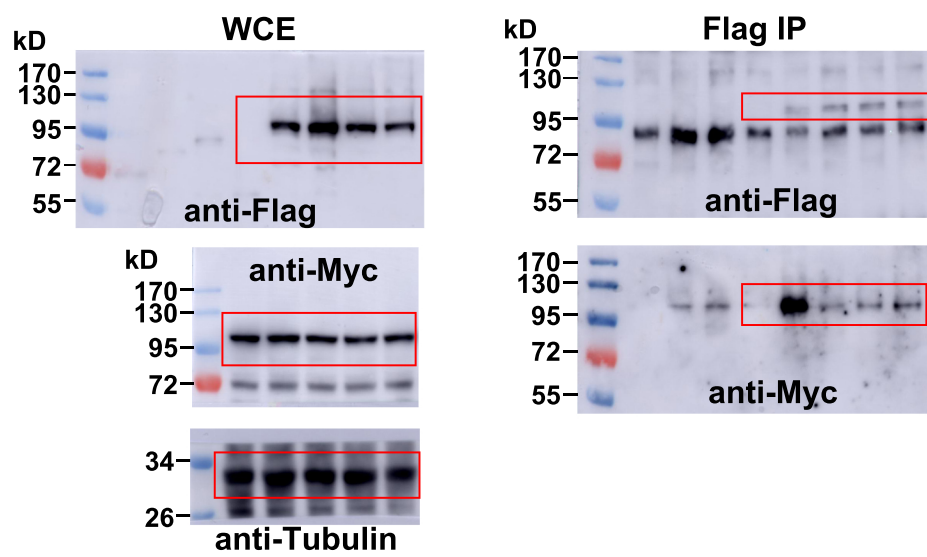

**Fig 2F**

Supplement: Supplementary file 1 [file LSA-2021-01106_SdataF2.pdf]
