# Supplementary material for: Ccq1–Raf2 interaction mediates CLRC recruitment to establish heterochromatin at telomeres
Source: Life Sci Alliance. 2021 Sep 7;4(11):e202101106. doi: 10.26508/lsa.202101106 (PMC8424379; doi:10.26508/lsa.202101106)
Supplement: Supplementary file 4 [file LSA-2021-01106_TableS1.docx]

**Table S1 Yeast strains used for this study.**

| **Strain name** | **Genotype** | **Source** |
| --- | --- | --- |
| TN9125 | *h^-^ ade6-M210 his3-D1 his3^+^::tel(1L)* | Moser *et al*, 2015 |
| ZW-2000 | *h^-^ ade6-M210 his3-D1 his3^+^::tel(1L) ccq1^+^::3FLAG-natMX* | TN9125 |
| ZW-2001 | *h^-^ ade6-M210 his3-D1 his3^+^::tel(1L) ccq1_1-439_::3FLAG-natMX* | TN9125 |
| ZW-2002 | *h^-^ ade6-M210 his3-D1 his3^+^::tel(1L) ccq1_1-500_::3FLAG-natMX* | TN9125 |
| ZW-2003 | *h^-^ ade6-M210 his3-D1 his3^+^::tel(1L) ccq1_1-583_::3FLAG-natMX* | TN9125 |
| ZW-2004 | *h^-^ ade6-M210 his3-D1 his3^+^::tel(1L) ccq1_1-439_-Clr3::3FLAG-natMX* | TN9125 |
| ZW-2005 | *h^-^ ade6-M210 his3-D1 his3^+^::tel(1L) ccq1_1-500_-Clr3::3FLAG-natMX* | TN9125 |
| ZW-2006 | *h^-^ ade6-M210 his3-D1 his3^+^::tel(1L) ccq1_1-439_-Clr4::3FLAG-natMX* | TN9125 |
| ZW-2007 | *h^-^ ade6-M210 his3-D1 his3^+^::tel(1L) ccq1_1-500_-Clr4::3FLAG-natMX* | TN9125 |
| ZW-2008 | *h^-^ ade6-M210 his3-D1 his3^+^::tel(1L) raf2^+^::13myc-hphMX6* | TN9125 |
| ZW-2009 | *h^-^ ade6-M210 his3-D1 his3^+^::tel(1L) raf2^+^::13myc-hphMX6 ccq1^+^::3FLAG-natMX* | ZW-2008 |
| ZW-2010 | *h^-^ ade6-M210 his3-D1 his3^+^::tel(1L) raf2^+^::13myc-hphMX6 ccq1^L511R^::3FLAG-natMX* | ZW-2008 |
| ZW-2011 | *h^-^ ade6-M210 his3-D1 his3^+^::tel(1L) raf2^+^::13myc-hphMX6 ccq1^V516R^::3FLAG-natMX* | ZW-2008 |
| ZW-2012 | *h^-^ ade6-M210 his3-D1 his3^+^::tel(1L) raf2^+^::13myc-hphMX6 ccq1^Y518R^-natMX* | ZW-2008 |
| ZW-2013 | *h^-^ ade6-M210 his3-D1 his3^+^::tel(1L) clr4^+^::3myc-hphMX6* | TN9125 |
| ZW-2014 | *h^-^ ade6-M210 his3-D1 his3^+^::tel(1L) clr4^+^::3myc-hphMX6 ccq1^+^::3FLAG-natMX* | ZW-2013 |
| ZW-2015 | *h^-^ ade6-M210 his3-D1 his3^+^::tel(1L) clr4^+^::3myc-hphMX6 ccq1^L511R^::3FLAG-natMX* | ZW-2013 |
| ZW-2016 | *h^-^ ade6-M210 his3-D1 his3^+^::tel(1L) clr4^+^::3myc-hphMX6 ccq1^V516R^::3FLAG-natMX* | ZW-2013 |
| ZW-2017 | *h^-^ ade6-M210 his3-D1 his3^+^::tel(1L) taz1^+^::13myc-hphMX6* | TN9125 |
| ZW-2018 | *h^-^ ade6-M210 his3-D1 his3^+^::tel(1L) taz1^+^::13myc-hphMX6 ccq1^+^::3FLAG-natMX* | ZW-2017 |
| ZW-2019 | *h^-^ ade6-M210 his3-D1 his3^+^::tel(1L) taz1^+^::13myc-hphMX6 ccq1^L511R^::3FLAG-natMX* | ZW-2017 |
| ZW-2020 | *h^-^ ade6-M210 his3-D1 his3^+^::tel(1L) rap1^+^::13myc-hphMX6* | TN9125 |
| ZW-2021 | *h^-^ ade6-M210 his3-D1 his3^+^::tel(1L) rap1^+^::13myc-hphMX6 ccq1^+^::3FLAG-natMX* | ZW-2020 |
| ZW-2022 | *h^-^ ade6-M210 his3-D1 his3^+^::tel(1L) rap1^+^::13myc-hphMX6 ccq1^L511R^::3FLAG-natMX* | ZW-2020 |
| ZW-2023 | *h^-^ ade6-M210 his3-D1 his3^+^::tel(1L) poz1^+^::13myc-hphMX6* | TN9125 |
| ZW-2024 | *h^-^ ade6-M210 his3-D1 his3^+^::tel(1L) poz1^+^::13myc-hphMX6 ccq1^+^::3FLAG-natMX* | ZW-2023 |
| ZW-2025 | *h^-^ ade6-M210 his3-D1 his3^+^::tel(1L) poz1^+^::13myc-hphMX6 ccq1^L511R^::3FLAG-natMX* | ZW-2023 |
| ZW-2026 | *h^-^ ade6-M210 his3-D1 his3^+^::tel(1L) tpz1^+^::13myc-hphMX6* | TN9125 |
| ZW-2027 | *h^-^ ade6-M210 his3-D1 his3^+^::tel(1L) tpz1^+^::13myc-hphMX6 ccq1^+^::3FLAG-natMX* | ZW-2026 |
| ZW-2027 | *h^-^ ade6-M210 his3-D1 his3^+^::tel(1L) tpz1^+^::13myc-hphMX6 ccq1^L511R^::3FLAG-natMX* | ZW-2026 |
