# Supplementary material for: Ccq1–Raf2 interaction mediates CLRC recruitment to establish heterochromatin at telomeres
Source: Life Sci Alliance. 2021 Sep 7;4(11):e202101106. doi: 10.26508/lsa.202101106 (PMC8424379; doi:10.26508/lsa.202101106)
Supplement: Supplementary file 5 [file LSA-2021-01106_TableS2.docx]

**Table S2 | Oligonucleotides used in this study.**

| **Oligonucleotides for qPCR** | | | | | | |
| --- | --- | --- | --- | --- | --- | --- |
| **DNA amplified** | **Primer** | | **Sequence (5′–3′)** | | | **Reference** |
| **Oligonucleotides for RT-PCR** | | | | | | |
| *act1^+^* | For | | AACCCTCAGCTTTGGGTCTT | | | Braun *et al*,  2011 |
|  | Rev | | TTTGCATACGATCGGCAATA | | |  |
| *his3^+^* | For | | GGTAAGCCTAGTAACGATGCC | | | van Emden et al, 2018 |
|  | Rev | | GTGCCAACAGTGATACGCAA | | |  |
| *TERRA* | For | | GAAGTTCACTCAGTCATAATTAATTGGGTAAC | | | Bah *et al*,  2012 |
|  | Rev | | GGGCCCAATAGTGGGGGCATTGTATTTGTG | | |  |
| *cen-dg* | For | | TGCTCTGACTTGGCTTGTCTT | | | Braun *et al*,  2011 |
|  | Rev | | CCCTAACTTGGAAAGGCACA | | |  |
| **Oligonucleotides for ChIP** | | | | | | |
| JK380/381  (100 bp from tel) | | For | | TATTTCTTTATTCAACTTACCGCACTTC | Harland *et*  *al*, 2014 | |
|  |  | Rev | | CAGTAGTGCAGTGTATTATGATAATTAAAATGG |  |  |
| Sg1906/1097  (20 bp from tel) | | For | | CGGCTGACGGGTGGGGCCCAATA | van Emden et al, 2018 | |
|  |  | Rev | | GTGTGGAATTGAGTATGGTGAA |  |  |
| Sg2038/2039  (744 bp from tel) | | For | | TTATTCACCCATACACACTACACC | van Emden et al, 2018 | |
|  |  | Rev | | GATGAATGGATTAAAAGGTGTTGG |  |  |
| Sg2102/2103  (1297 bp from tel) | | For | | ATCTACTCCAATATAGTCCTCTGC | van Emden et al, 2018 | |
|  |  | Rev | | GATAATGGATGGAGGTAAGAGAGG |  |  |
| Sg2141/2142  (2012 bp from tel) | | For | | TACTCCAACACACTCAATACATACC | van Emden et al, 2018 | |
|  |  | Rev | | AAGTAGGAGAATGAAGAAGTAATCAAAG |  |  |
| Sg2106/2107  (2275 bp from tel) | | For | | TTATATTCCTGCATCCCAACACAT | van Emden et al, 2018 | |
|  |  | Rev | | AAAGAAGATAAAAGCAGGGGACTA |  |  |
| Sg2139/2149  (2758 bp from tel) | | For | | TCGTTAACAACATTTAACGATTACTCG | van Emden et al, 2018 | |
|  |  | Rev | | ACGTTTGTTGAGTGATATGTCGTCG |  |  |
